# Probiotics and the intestinal tight junction barrier function

**DOI:** 10.3389/fcell.2025.1671152

**Published:** 2025-12-01

**Authors:** Megan M. Ferris, Ligia Subitoni Antonio, Rana Al-Sadi

**Affiliations:** Frederick F. Paustian IBD Center, Division of Gastroenterology and Hepatology, Department of Internal Medicine, University of Nebraska Medical Center, Omaha, NE, United States

**Keywords:** Probiotics, tight junctions, intestinal permeability, intestinal inflammation, probiotic signaling

## Abstract

Disruption of the intestinal epithelial tight junction (TJ) barrier is a key pathogenic factor in numerous gastrointestinal (GI) disorders, including inflammatory bowel disease, irritable bowel syndrome, necrotizing enterocolitis, and enteric infections. The gut microbiota plays a pivotal role in regulating epithelial integrity, and emerging evidence highlights the therapeutic potential of probiotics in preserving or restoring TJ barrier function. This review summarizes the current literature on the protective effects of probiotics in modulating intestinal epithelial TJ barrier function. Specific strains of *Lactobacillus*, *Bifidobacterium*, *Escherichia coli* Nissle 1917, *Bacillus subtilis,* and *Saccharomyces boulardii* have been shown to enhance barrier integrity in cell culture, animal models, and in some clinical settings. These probiotics exert their effects through diverse mechanisms, including the upregulation of TJ proteins (e.g., occludin, claudins, ZO-1), suppression of proinflammatory cytokines, inhibition of NF-κB, myosin light chain kinase (MLCK) and MAPK signaling pathways, and activation of host pattern recognition receptors such as TLR-2 and PPARγ. Moreover, several studies highlight the strain-specific nature of these effects, underscoring the importance of identifying and characterizing individual probiotic strains for therapeutic use. Taken together, the data reviewed here support the potential of probiotics as adjunctive or preventive therapies targeting epithelial barrier dysfunction in a range of GI diseases. However, further mechanistic studies, clinical trials, and standardization of probiotic formulations are needed to translate these findings into effective, personalized interventions. This review highlights both the promise and complexity of probiotic-mediated intestinal barrier regulation and provides new insight for future research in this rapidly evolving field.

## Introduction

**FIGURE 1:**
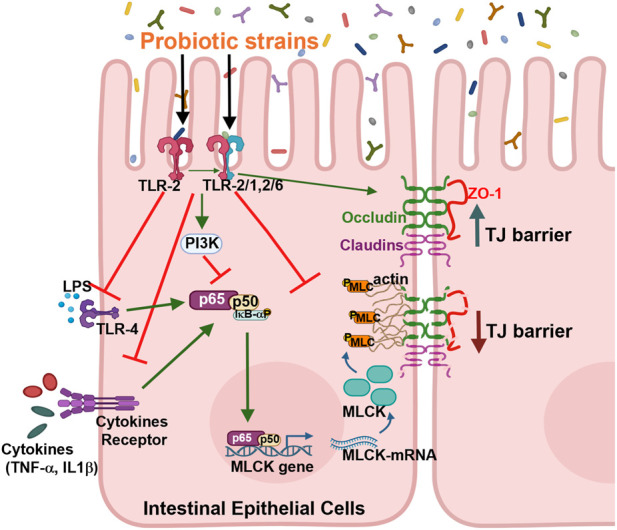
Schematic illustration presents aconceptual model summarizing probiotic strains-mediated signaling pathways implicated in the regulation of tight junction barrier.

Dysregulated mucosal permeability and epithelial integrity plays a crucial role in the pathophysiology of a variety of gastrointestinal (GI) disorders, which include pathogen infection (De Winter et al., 2012), inflammatory bowel disease (IBD) (Caradonna et al., 2000; Han, 2010), irritable bowel syndrome (IBS) (Ohman and Simren, 2010), obesity and the metabolic syndrome (Greenwood-Van Meerveld et al., 2017) and necrotizing enterocolitis (NEC) (Goldman, 2000; Halpern et al., 2006; Rosenfeldt et al., 2004). A compromised epithelial tight junction (TJ) barrier is a hallmark of these conditions, allowing luminal antigens, toxins, and microbes to translocate across the epithelium and trigger inappropriate immune responses. Tight junctions are multiprotein complexes composed of transmembrane proteins (e.g., claudins and occludin), cytoplasmic adaptor proteins (e.g., Zonula Occludens (ZO-1)), and regulatory scaffolding elements (Capaldo et al., 2017). They are the apical-most intercellular junctional complexes in the intestinal epithelium and play a central role in maintaining barrier integrity (Suzuki, 2013; Fasano and Nataro, 2004; Gasbarrini and Montalto, 1999). Canonically, TJs regulate paracellular permeability by selectively controlling the passage of ions, solutes, and water between epithelial cells, thereby preserving transepithelial gradients (Suzuki, 2013). Beyond barrier function, TJs maintain cell polarity, restrict the diffusion of membrane proteins and lipids, and contribute to mucosal immune defense by limiting luminal antigen and microbial translocation (Paradis et al., 2021; Suzuki, 2020). Disruption of TJ structure or regulation is a key pathological feature in many GI disorders. Probiotics refer to “live microorganisms which when administered in adequate amounts and proper combination confer a health benefit to the administered organism, such as humans” (Pineiro and Stanton, 2007). Commonly recognized intestinal probiotics include *Lactobacillus*, *Bifidobacterium*, *Streptococcus*, and a few *Escherichia coli* strains (Eckburg et al., 2005). These bacteria have long been proven to regulate intestinal epithelial function by facilitating the formation of mucous layers, secreting antibacterial factors, competitive adhesion to intestinal epithelial cells (Artis, 2008), and increasing TJ formation (Rook et al., 2017). Beyond their studied role in IBD, probiotics are increasingly being marketed and prescribed for a variety of GI disorders, including IBS and other functional bowel disorders (Ceccherini et al., 2022; Dai et al., 2014). In IBS, probiotics have been investigated for their potential to improve abdominal pain, bloating, stool consistency, and overall quality of life, largely by modulating gut motility, visceral sensitivity, and microbial composition (Ceccherini et al., 2022). Similarly, in functional bowel disorders such as functional dyspepsia or chronic constipation, probiotic strains have been explored for their capacity to normalize bowel habits and reduce symptom severity (Tziatzios et al., 2023). These emerging applications reflect a growing recognition that probiotics may exert benefits across a broader spectrum of gut health conditions, extending beyond inflammation-driven pathologies to disorders characterized by altered dysbiosis and barrier dysfunction. Thus, numerous studies using cell models, animal models, and patient populations have evaluated the protective and preventive effects of probiotics in GI functions and disorders by clinical parameters, such as TJ proteins and TJ barrier function (Gorbach, 2000; Isolauri et al., 2001). A healthy intestinal TJ barrier is selectively permeable, permitting passage of essential nutrients and water, while restricting absorption of toxins, antigens, and pathogens. Intestinal permeability is affected by multiple factors, including proinflammatory cytokines [for example, tumor necrosis factor alpha (TNF-α) and interferon gamma (IFN-γ)] (Ma et al., 2004), epithelial apoptosis and various exogenous factors, such as alcohol (Swanson et al., 2010), high fat diet (HFD) (Ma et al., 2008) and non-steroidal anti-inflammatory drugs (Oshima et al., 2008). In addition, defects in intestinal permeability have been associated with alterations in the gut microbiota composition and function (Wu and Lewis, 2013). For instance, some pathogens impair the intestinal epithelial TJ barrier and cause acute inflammation (O'Hara and Buret, 2008). Chronic inflammation compromises nutrient bioavailability and allows luminal antigens to stimulate underlying immune cells in various irritable bowel diseases, including celiac disease, Crohn’s disease (CD), diabetes, and food allergy (Suenaert et al., 2002; Ventura et al., 2006). The mechanism by which these multiple factors affect intestinal permeability is diverse but is often associated with alteration of TJ protein expression (Mankertz and Schulzke, 2007), increase in myosin light chain kinase (MLCK) mediated opening of intestinal epithelial TJ barrier (Du et al., 2023; McCole, 2022), and activation of nuclear factor kappa-light-chain-enhancer of activated B cells (NF-κB) (Arrieta et al., 2006). A critical mechanism of TJ regulation involves the actomyosin cytoskeleton, particularly through MLCK-mediated phosphorylation of myosin II regulatory light chain (MLC2) (Al-Sadi et al., 2011; Ma et al., 2000a; Ma et al., 2000b; Ye et al., 2006). This signaling event increases actomyosin contractility, leading to peri-junctional ring contraction and subsequent TJ opening (Ma et al., 2000a; Ma et al., 2000b). Elevated MLCK activity has been consistently associated with barrier disruption under inflammatory conditions (Al-Sadi et al., 2011; Ma et al., 2000a; Al-Sadi et al., 2021a; Al-Sadi et al., 2012; Al-Sadi et al., 2008; Boivin et al., 2007; Ma et al., 2005; Ma et al., 1999; Wang et al., 2005). Interestingly, several studies indicate that probiotics may modulate MLCK signaling, either by downregulating MLCK expression or by interfering with upstream activators such as NF-κB and proinflammatory cytokines (Abdulqadir et al., 2024; Haque et al., 2024; Huang et al., 2020; Ren et al., 2022; Yi et al., 2018). Herein, we review the literature on the role of probiotics in intestinal epithelial integrity and the beneficial effects of probiotics in the alleviation of GI disorders and present the main intracellular mechanisms involved (Figure 1; Supplementary Table 1).

## Lactobacillus

*Lactobacillus* spp. is one of the most widely used probiotics in the production of fermented foods derived from both animals (for example, milk and meat) and plants (for example, vegetables and cereal) (Giraffa et al., 2010). The genus *Lactobacillus* comprises a large heterogeneous group of Gram-positive, facultative anaerobic bacteria which include *L. acidophilus*, *L. rhamnosus, L. fermentum, L. casei, L. plantarum, L. helveticus, L. reuteri*, to name a few (Mu et al., 2018). Besides its role in food fermentation, the *Lactobacillus* genus is also a healthy component in the GI system of humans and animals in variable amounts depending on the species, age of the host, or location within the gut (Duar et al., 2017).

Animal studies and preclinical results have shown that *lactobacilli* may help in the prevention and treatment of numerous GI disorders. Among these disorders are enteric infections, antibiotic-associated diarrhea, NEC in preterm neonates, IBD, colorectal cancer, and IBS. Multiple studies have confirmed that *lactobacilli* play an important role in the management of GI disorders by maintaining epithelial barrier integrity both *in vitro* and *in vivo*. Various mechanisms of this function include modulation of the cytoskeleton, induction of mucus production, and phosphorylation of TJ proteins, which result in the enhancement of TJ function and the immune response, as well as the prevention of epithelial cell apoptosis (Yoda et al., 2014; Zhang Z. et al., 2018).

Different strains of *Lactobacillus* and their roles in maintaining healthy TJ barrier are summarized below.

### *Lactobacillus amylophilus* D14

Yu et al. have shown that healthy human colorectal adenocarcinoma cells (Caco-2) treated with *Lactobacillus amylophilus* D14 showed no change in TJ permeability. However, when Caco-2 cells were damaged by pathogenic, enterogenic *E. coli* K88 or *Salmonella typhimurium (S. typhi)* SL1344, *Lactobacillus amylophilus* D14 protected TJ proteins, ZO-1, claudin-1, E-cadherin, and the TJ barrier function, which was mediated by the reduction of phosphorylated Extracellular signal-Regulated Kinase (ERK) and secretion of Interleukin-8 (IL-8) (Yu et al., 2012).

### Lactobacillus acidophilus

*Lactobacillus acidophilus* contains unique surface layer proteins, or Slp, which has been confirmed to exhibit multiple biological properties, including the ability to competitively bind to intestinal adhesion sites, to inhibit apoptosis, and to reduce lipopolysaccharide (LPS)-induced inflammation (Meng et al., 2015; Meng et al., 2017; Wang H. et al., 2018). Wang et al. showed that Caco-2 cells treated with Slp depicted enhanced intestinal epithelial TJ barrier by increasing trans epithelial resistance (TER), decreasing dextran flux *via* restoration of ZO-1 and occludin, and reducing the secretion of IL-8. [TER is a quantitative measure of ionic conductance across epithelial monolayers and is widely used as a marker of TJ integrity. Increases in TER reflect a tightening of the paracellular pathway and enhanced barrier function, whereas decreases indicate TJ barrier compromise (Abdayem et al., 2015; Moyes et al., 2007). Because TER provides a real-time and noninvasive assessment, it is often combined with permeability assays (e.g., dextran flux) to strengthen conclusions about TJ modulation.] Furthermore, the addition of Slp inhibits the TNF-α induced increase in TJ permeability, apoptosis and activation of NF-κB (Wang et al., 2019). A recent report tested more than 20 species and strains of probiotics and demonstrated that *L. acidophilus* induced a marked enhancement of intestinal epithelial TJ barrier function in Caco-2 monolayers. These findings also demonstrated that a specific strain of *L. acidophilus*, LA1, produced a near-doubling effect (90%–100%) compared with LA2 (50%), whereas LA3 did not affect intestinal epithelial TJ barrier function (Al-Sadi et al., 2021b). The LA1 effect on TJ barrier function was shown to be mediated by the Toll-like receptor-2/TLR-1 and TLR-2/TLR-6 heterodimeric complex and had a protective effect against dextran sodium sulfate (DSS)-induced colitis in wild-type mice, where these effects were abolished in TLR-2 knockout mice (Al-Sadi et al., 2021b). Most recently, Haque et. al illustrated how the intracellular mechanisms of LA1 are cell-specific. As expected, LA1 exposure to immune cells produced a TLR-2 and MyD88-dependent activation of NF-κB p50/065. Interestingly, LA1 exposure to intestinal epithelial cells inhibited the TNF-α-induced increase in TJ permeability via a TLR-2 dependent and MyD88-independent activation of PI3K, leading to the inhibition of NF-κB p50/60 and MLCK activity. (Haque et al., 2024). These findings suggested that the LA1 mechanism of action differs in intestinal epithelial cells from that in immune cells. Another study showed that a specific strain of *L. acidophilus* W37 enhanced TER by 15% in Caco-2 compared to *L. brevis* and *L. casei,* which had no effect on TER. A microarray analysis demonstrated that *L. acidophilus* W37 has the capacity to enhance barrier function *via* upregulation of TJ proteins occludin, claudin-4, claudin-15 and claudin-16. Furthermore, *L. acidophilus* W37 attenuated *S. typhi*-induced decrease in TER and IL-8 secretion in Caco-2 (Lepine et al., 2018). Another study showed that secreted metabolites from a strain of *L. acidophilus* caused a mild increase in TER (25%) and a decrease in dextran flux in Caco-2. Conditioned media of *L. acidophilus* prevented the IL-1β induced increase in TJ permeability by inhibiting NF-κB activation and normalizing TJ proteins occludin and claudin-1 (Guo et al., 2017). A previous report showed that heat-inactivated *L. acidophilus* and its supernatant protected the aspirin-induced damage in TJ barrier function in human colon cancer cells (HT-29) by modulating the expression and localization of ZO-1 (Montalto et al., 2004). Wu et al. showed that *L. acidophilus* prevented *Salmonella-*induced colitis in mice and preserved the colonic barrier by inhibiting the Notch transcription factors (Wu et al., 2018). Other studies showed that live *L. acidophilus* LAB20, increased TER by 50% and strengthened the intestinal barrier function in Caco-2. The live LAB20 also prevented the LPS-induced IL-8 production of HT-29 cells. However, the freeze-dried LAB20 could not exert the same effect on HT-29 (Kainulainen et al., 2015). Taken together, the beneficial effects of LA can largely be observed with the live probiotic, its supernatant, or denatured form, but its effects are strain and cell-specific.

### Lactobacillus casei

The effect of *Lactobacillus casei* subsp. *rhamnosus* Lcr35 on the intestinal epithelial TJ barrier was examined in the presence of *Salmonella* LPS. In this study, Caco-2 cells co-cultured with peripheral blood mononuclear cells (PBMCs) in the basolateral compartment were treated with *Salmonella* LPS, and then incubated with Lcr35 for 1, 6, 24 or 48 h. Compared to cells treated with LPS alone, apical co-incubation with Lcr35 showed an increase in TER by 87% after 48 h of incubation. Also, Lcr35 significantly inhibited the basolateral secretion of IL-8 in the Caco-2/PBMC co-culture (Fang et al., 2010). A recent study investigated the protective effect of *L. casei* on intestinal barrier dysfunction and the possible relationship with mast cells. *Lactobacillus casei* was shown to increase the TER in porcine intestinal epithelial cells by 60% compared to control cells; and decrease permeability to dextran by more than 80%. *Lactobacillus casei* alleviated the intestinal epithelial barrier dysfunction in porcine jejunal epithelial cell line (IPEC-J2) and mice infected with enterotoxigenic *E. coli* K88 (ETEC K88) by preventing the pathogen-induced downregulation of TJ proteins, occludin, claudin-1 and ZO-1, and TLR-2 and TLR-4 expression (Xu et al., 2020).

Previous studies showed that lysates of *L. casei* DN-114 001 can protect mice from DSS-induced intestinal inflammation, thus conferred a health benefit. *Lactobacillus casei* inhibited the DSS-induced increase in permeability to dextran, which was mediated by a decrease in inflammatory cytokines TNF-α and IFN-γ, and by changing the composition of gut microbiota (Zakostelska et al., 2011). Live bacteria of *L. casei*, but not its supernatants, prevented the TNF-α and IFN-γ-induced drop in TER. This study also showed that *L. casei* prevented the cytokine-induced increase in permeability *via* MAPK/PI3kinase/Akt pathway phosphorylation, increased TLR-2 expression, and preservation of ZO-1 protein expression in Caco-2 (Eun et al., 2011). Prassol et al. found similar protective effects of *L. casei* DN-114 001 against enteropathogenic *E. coli* (EPEC) infection in T84 cells (Parassol et al., 2005).

### Lactobacillus plantarum

A human study by Mujagic *et al.* demonstrated that three strains of L*. plantarum* (*L. plantarum* WCFS1, CIP104448, and TIFN101) did not prevent the indomethacin-induced increase in small intestine permeability measured by urine Lactulose-rhamnose ratio (Mujagic et al., 2017). However, *L. plantarum* TIFN101 modulated gene transcription pathways related to tight- and adhesion junction protein synthesis and degradation, including actinin alpha-4, metalloproteinase-2 (Mujagic et al., 2017). Another study by Karczewski *et.al*. showed that *L. plantarum* WCFS1 induced the expression of ZO-1 and occludin in human small intestinal tissues, and ∼10% increase in TER *via* activation of TLR-2/NF-κB pathway in Caco-2 (Karczewski et al., 2010). In addition, *L. plantarum* showed a protective effect against phorbol 12,13-dibutyrate (PDBu)-induced drop in TER in Caco-2, which was similar to the protective effect of TLR-2-synthetic agonist, Pam (Han, 2010)Cys-SK4 (PCSK) (Karczewski et al., 2010). Other investigators using IPEC-J2 showed that *L. plantarum* ZLP001 significantly inhibited the ETEC-induced increase in gut permeability to dextran. In addition, *L. plantarum* ZLP001 pretreatment restored the TJ proteins, claudin-1, occludin, and ZO-1, and downregulated proinflammatory cytokines, IL-6 and IL-8, and TNF-α expression and secretion caused by ETEC. *Lactobacillus plantarum* ZLP001 also significantly increased the expression of the host defense porcine beta-defensin 2 (pBD2) and protegrins peptides (PG1-5). Furthermore, *L. plantarum* ZLP001 treatment affected piglet fecal microbiota, suggesting that *L. plantarum* ZLP001 enhanced the intestinal barrier by strengthening epithelial defense functions and modulating gut microbiota (Wang J. et al., 2018; Zhang W. et al., 2018).

Another study showed that the exopolysaccharides extracted from *L. plantarum* NCU116 (EPS116) attenuated DSS-induced colitis and promoted epithelial barrier function and the expression of TJ proteins ZO-1 and occludin both *in vivo* and *in vitro* in a signal transducer and activator of transcription 3 (STAT3)-dependent manner. They also showed that knocking-down of STAT3 in Caco-2 with EPS116 treatment led to decreased expression of occludin and ZO-1 and increased intestinal permeability, suggesting that EPS116 inhibited intestinal inflammation *via* regulating intestinal epithelial barrier function (Zhou et al., 2018). Moreover, *L. plantarum* has been shown to protect intestinal epithelial barrier function from ETEC K88 infection in NCM460 cells derived from normal human colon mucosal epithelium. *Lactobacillus plantarum* enhanced IL-22 production in natural killer (NK) cells that protected the intestinal TJ barrier of despite infection with ETEC (Qiu et al., 2017). Other investigators showed that a specific strain of *L. plantarum,* DSM 2648 caused an increase in Caco-2 TER by almost 235% compared to control. In addition, *L. plantarum* DSM 2648 a2648 attenuated the effect of EPEC O127:H6 (E2348/69) on TER by 98.75% and inhibited EPEC adherence by 80.18% (Anderson et al., 2010a). In the same study, *L. rhamnosus* HN001 was shown to increase Caco-2 TER by 148% compared to control. *Lactobacillus plantarum* HY7714 has been reported to have a protective effect on TNF-α treated Caco-2 monolayers by preserving the TJ proteins ZO-1, claudin-1 and occludin. Further, *L. plantarum* HY7714 prevented the TNF-α induced increase in the mRNA levels of transcription factor from Ets family (Elk-1), NF-κB, and MLCK, implying that HY7714 improves intestinal barrier integrity and is a potential therapeutic agent to treat dysfunctions derived from TJ defects (Nam et al., 2019). Other studies showed that a specific strain of *L. plantarum* MB452 caused a dose-dependent increase in Caco-2 TER by 60% compared to control group over 10-h experimental period. The effect of *L. plantarum* on the TJ barrier function was due to the enhancement of TJ protein expression of ZO-1 and occludin (Anderson et al., 2010b). A recent study demonstrates that *Lactobacillus plantarum* LR002 (LR) improves ulcerative colitis (UC) progression in mice by activating peroxisome proliferator-activated receptor gamma (PPARγ) signaling and inhibiting the Mitogen-Activated Protein Kinase (MAPK)/NF-κB pathway (Zang et al., 2024). In DSS-induced colitis, LR restores the expression of TJ proteins (ZO-1, Occludin, Claudin-3). In DSS-induced colitis, LR was found to restore the expression of TJ proteins, reduce proinflammatory factors, lower oxidative stress, modify the intestinal flora, and restore short-chain fatty acid (SCFA) levels (Zang et al., 2024).

### Lactobacillus rhamnosus

Earlier study by Gupta *et al.* evaluated the efficacy of *L. rhamnosus* (LGG) in children with CD and showed a significant improvement in intestinal permeability (as measured by a double sugar permeability test in the urine), and a significant improvement in the activity of the CD patients. However, this improvement was not sustained at 24-week follow-up (Gupta et al., 2000). A recent report found that a specific strain of *L. rhamnosus,* CNCM I-3690, can protect intestinal barrier functions in a mouse inflammation model. CNCM I-3690 prevented the TNF-α induced decrease in TER in Caco-2, and physically modulated goblet cells and the mucus layer. Furthermore, mice colonic transcriptome analysis revealed that CNCM I-3690 enhanced the expression of genes related to healthy gut permeability: motility and absorption, cell proliferation; and protective functions by inhibiting endogenous proteases (Martin et al., 2019). Various studies provide *in vitro* evidence that supports the protective effect of the live LGG probiotic on the TJ barrier against inflammatory and pathogenic conditions. Donato et *al.* challenged Caco-2 cells with TNF-α and IFN-γ and posited that the live LGG protective effect was mediated by the inhibition of the NF-KB and ERK1/2 pathway (Donato et al., 2010). Moreover, other strains of probiotics used in these studies (*L. farciminis* and *L. plantarum*) did not provide the same barrier-protective effect, even at higher concentrations. A similar study by Han et al. with human enteroids and colonoids demonstrated that live LGG treatment prevented the IFN-γ-induced downregulation of TJ barrier proteins (Han et al., 2019). Previous study showed that LGG prevented the gliadin-induced increase in Caco-2 TJ permeability as demonstrated by the rapid decrease in TER, a significant increase in lactulose paracellular transport, and a slight downregulation in ZO-1 and occludin expression but not claudin-1. The co-administration of viable LGG, LGG-HK (heat-killed) and LGG-CM (conditioned media) with gliadin significantly restored TJ barrier function in Caco-2 (Orlando et al., 2014).

Additionally, pretreatment of polarized Madin-Darby canine kidney (MDCK) and human colonic adenocarcinoma (T84) cells with live LGG attenuated the EHEC O157:H-induced drop in TER and increase in TJ permeability. In addition, LGG protected epithelial monolayers against Enterohemorrhagic *E. coli* (EHEC)-induced redistribution of the claudin-1 and ZO-1. In contrast to the effects seen with the live probiotic, heat-inactivated *LGG* did not affect disruption of the barrier function. Collectively, these findings provided *in vitro* evidence that treatment with the probiotic *LGG* could be an effective treatment to prevent injury of the TJ barrier induced by bacterial entero-pathogens (Johnson-Henry et al., 2008). Different studies showed that a strain of *L. rhamnosus* (MTCC-5897) by itself did not affect the Caco-2 TER or permeability, but protected against the *E.coli*-induced disruption of TJ barrier function, which was mediated by an increase and re-distribution of ZO-1 and occludin (Bhat et al., 2019). *L. rhamnosus* OLL2838 was found to effectively suppress barrier impairment and increased IL-8 secretion induced by TNF-α in Caco-2, however, the conditioned medium from OLL2838 did not show any effect on barrier functions. Oral treatment with both live and heat-killed OLL2838 protected against the increase in mucosal permeability associated with DSS-induced colitis in mice. This was associated with increased expression of ZO-1 and MLCK in intestinal epithelial cells isolated from mice treated with heat-killed OLL2838 (Miyauchi et al., 2009).

### Lactobacillus helveticus

Ho *et al.* studied the effect of live *L. helveticus* ASCC 511 (LH511) on the integrity of the intestinal epithelial TJ barrier function in IPEC-J2 cells and found that its cell growth-promoting effects were increased when incubated with fermented milk (FM) and citrulline. This combination effectively enhanced the intestinal epithelial TJ barrier under normal conditions, reduced the adhesion of EHEC and EIEC, and restored barrier damage induced by LPS. These effects were mediated by the activation of TLR-2 and TLR-9 and the suppression of TLR-4 (Ho et al., 2020).

### Lactobacillus fermentum

Unlike other probiotic strains, a strain of *L. fermentum,* AGR1487, was shown to reduce TER in a dose-dependent manner compared to controls in Caco-2. Although *L. fermentum* AGR1487 had a negative effect on TER, its supernatant caused a 34% increase in Caco-2 TER compared to control media. On the other hand, *L. fermentum* AGR1485 or its supernatant did not affect TER compared to control media in Caco-2. In addition, *L. fermentum* AGR1487 but not *L. fermentum* AGR1485 caused an increase in mannitol flux across the Caco-2 monolayers. Taken together, these results suggested that despite being in the species, *L. fermentum* AGR1487 increased the expression of genes and abundance of tubulins and microtubule-associated proteins that have been implicated in reducing the disassembly of TJs, thus affecting the intestinal TJ barrier function negatively (Anderson et al., 2013).

### Lactobacillus reuteri

Recent study showed that *L. reuteri* FN041, a strain isolated from human breast milk, attenuated HFD-induced increase in mouse intestinal permeability. *Limosilactobacillus reuteri* FN041 induced effect on the intestinal TJ barrier was accompanied by the inhibition of HFD-induced increase production in LPS, TNF-α and IL-16. In addition, *L. reuteri* FN041 prevented the HFD-induced downregulation of occludin, ZO-1, claudin-6, and claudin-7 in mouse intestinal tissues (Li et al., 2019). It has been shown that the beneficial effect of *L. reuteri* FN041 was due to the reduction in short-chain fatty acid production by the gut microbiota (Li et al., 2019). Another study has shown that pretreatment with *L. reuteri* isolated from the feces of a healthy weaned piglet decreased the intestinal permeability to dextran in ETEC K88-infected IPEC-1 but had no effect when used alone. That protective effect of *L. reuteri* was associated with preventing the ETEC-induced decrease in ZO-1 and occludin expression in a MLCK-dependent manner (Yi et al., 2018). A similar study showed the protective effect of *L. reuteri* against ETEC K88-induced increase in IPEC-1 cells by inhibiting the destruction of ZO-1. Interestingly, these studies showed that treatment with *L. reuteri* alone caused a 25% decrease in dextran permeability in IPEC-1 cells (Wang et al., 2016). Different strain of live *L. reuteri*, 15007, was shown to increase the TER in IPEC-J2 by only 8% after 10 h of treatment and that was associated with a mild increase in expression of claudin-1, occludin, and ZO-1. In a separate set of experiments, the studies showed that live bacteria and supernatants of *L. reuteri* 15007 prevented the LPS-induced downregulation of the TJ proteins, claudin-1, occludin and ZO-1, and suppressed the LPS-induced overexpression of TNF-α and IL-6 (Yang et al., 2015). Another strain of *L. reuteri*, P43-HUV, has also been found to cause ∼10% increase in the TER of IPEC-J2, and prevent the ETEC-induced increase in TJ permeability. The authors also showed the beneficial effect of *Lactobacillus johnsonii* in preventing the ETEC-induced increase in TJ permeability; however, *L. johnsonii* alone did not affect the barrier in IPEC-J2 cells. The protective effect of these two probiotic strains was mediated by an increase in heat-shock protein (HSP)-27 and ZO-1 (Liu et al., 2015).

### Lactobacillus salivarius

*L. salivarius* SMXD51 has been shown to increase in TER by 20% after 24 h treatment, and a protective effect against *Pseudomonas aeruginosa* PAO1-induced decrease in TER in Caco-2. The probiotic and beneficial effect of *L. salivarius* SMXD51 was mediated by an upregulation of the F-actin cytoskeleton (Miyauchi et al., 2012; Messaoudi et al., 2012). Other intensive study by Miyauchi et. al demonstrated the efficacy of different *L. salivarius* strains in protecting against hydrogen peroxide (H_2_O_2_)-induced barrier impairment in Caco-2 cells. After evaluating 33 strains of *L. salivarius*, the authors showed that the effective strains UCC118 and CCUG38008 attenuated H_2_O_2_-induced disassembly and re-distribution of TJ proteins [ZO-1, occludin, claudin-1, and Junctional Adhesion Molecule (JAM)] in ERK1/2 phosphorylation-dependent manner, but the ineffective strain AH43324 did not (Miyauchi et al., 2012).

## *E.coli* Nissle 1917 (EcN)

Hering et al. showed that the live bacteria and supernatant, but not heat-killed bacteria, of EcN caused around 40%–75% increase in TER in HT-29 and Caco-2, and a 40% decrease in permeability to mannitol. This was attributed to the TcpC (Toll/IL-1 receptor (TIR) domain-containing protein C) protein of EcN. These studies indicated that the enhancing effect of EcN on the TJ barrier was mediated by TcpC-induced phosphorylation of protein kinase C delta (PKCζ) and ERK1/2 and increase in protein expression of TJ barrier forming claudin-14 (Hering et al., 2014).Wang et al. showed that supernatant of EcN prevented 5-fluoro-1H-pyrimidine-2,4-dione (5-FU) induced decrease in TER values in IEC-6 cells (Wang et al., 2014). Guo et al. demonstrated that the supernatant of EcN protected the TJ barrier function in sepsis both *in vivo* and *in vitro* by inhibiting the increased NF-κB-mediated activation of the MLCK-P-MLC signaling pathway. EcN supernatant, when co-administered with TNF-α and IFN-γ, significantly alleviated barrier dysfunction by improving TER (Guo et al., 2019). Recent studies by Alvarez et al. showed the protective effect of outer membrane vesicles and soluble factors of EcN on TJ barrier in EPEC-infected T-84 and Caco-2. The protective effect of EcN was associated with the preservation of occludin and claudin-14 expression (Alvarez et al., 2019). In a DSS-induced colitis mouse model, EcN prevented the DSS-induced increase in TJ permeability, and which was associated with the preservation of ZO-1 expression (Ukena et al., 2007).

## Bacillus subtilis

Rhayat *et al.* showed the effect of three different strains of *B. subtilis* on TJ barrier function in Caco-2. *Bacillus subtilis* 29784 caused a 50% increase in TER, whereas the others had no or detrimental effects. The effect of *B. subtilis* 29784 on TJ barrier was associated with an increase in ZO-1, occludin, and cluadin-1 in Caco-2. In addition, *B. subtilis* showed strain-specific inhibition of the IL-1β-induced activation of NF-κB and significant decrease in IL-8 production and reduction of the upregulation of inducible nitric oxide synthase (iNOS) protein levels in IL-1β treated cells (Rhayat et al., 2019).

## Bifidobacterium

Among the various probiotic bacteria reported to date, *Bifidobacterium* spp., is one of the most widely studied and utilized probiotic bacteria. The genus *Bifidobacterium* belongs to the phylum Actinobacteria, and currently comprises 80 (sub) species, which are distributed across different ecological niches including GI tract and oral cavity of human (Turroni et al., 2011; Ventura et al., 2007). They are Gram-positive, non-motile, non-sporulating anaerobic bacilli. *Bifidobacterium* spp. are the first to colonize the human intestine, a phenomenon driven by the bifidogenic activities of certain mother milk derived oligosaccharides (Garrido et al., 2013). Consequently, they account for nearly 80% of microorganisms in the intestinal tract of breastfed infants (Soto et al., 2014). *B. breve, B. bifidum, B. longum,* and *Bifidobacterium infantis* are the commonly detected bacteria at the infant stage, with *B. bifidum* being the most prominent species, followed by *B. breve, B. longum* and *B. infantis* (Turroni et al., 2012; Kolacek et al., 1996). As age progresses, the overall concentration of *Bifidobacterium* decreases, but remains relatively stable (2%–14%) throughout adulthood and decreases again in old age (Odamaki et al., 2016). Commonly identified species in the adult gut include *B. adolescentis* and *B. catenulatum*, followed by *B. longum* and *B. bifidum* (Chaplin et al., 2015; Turroni et al., 2014). However, there is no absolute infant *versus* adult division of bifidobacterial species.

Bifidobacteria have been commercially exploited as probiotic agents due to their well-established health benefits and GRAS (Generally Recognized As Safe) status (Picard et al., 2005). The claimed homeostatic and health-promoting activities exerted by bifidobacteria are numerous, which include establishment of a healthy microbiota in preterm infants (Wang et al., 2007), protection against pathogens (Arunachalam et al., 2000), enhancement of intestinal gut barrier (Chichlowski et al., 2012; Furrie, 2006), promoting an anti-inflammatory environment through modulation of host immune response (Lopez et al., 2011), production of vitamins and short chain fatty acids, digestion of plant oligo- and poly-saccharides, and suppressing the production of potentially toxic and carcinogenic metabolites (Sela et al., 2008). Being an innate member of the human gut, *Bifidobacterium* has been proven to be essential for maintaining the intestinal epithelial barrier integrity (Bergmann et al., 2013). Indeed, a recent study cataloging the microbiota of UC patients by 16S rRNA microbial profiling revealed a substantial decrease of bifidobacteria, notably *B. bifidum*, suggesting that this taxon plays a biological role in the etiology of UC and also highlighted the importance of *B. bifidum* as a microbial biomarker for UC (Duranti et al., 2016).

Previous studies have shown that pretreatment of human intestinal epithelial cell lines (Caco-2, HT-29 and T-84) with bifidobacteria species confers protective effects against TJ barrier impairment induced by various factors. The protective effects are mediated through upregulation of expression of TJ proteins (notably occludin and ZO-1). Furthermore, bifidobacteria species also modulate various protein kinase signaling pathways, leading to the phosphorylation of TJ proteins, which can either promote TJ formation or redistribution and complex stabilization (Chichlowski et al., 2012; Hsieh et al., 2015; Ling et al., 2016). Another study demonstrated that *Bifidobacterium bifidum* BB1 caused tightening of the intestinal TJ barrier in a strain-specific manner by increasing TER up to 50%–80% in Caco-2 monolayers. The BB-enhancement was shown to be mediated by BB1 attachment to the TLR-2 complex on the apical surface of enterocytes, leading to the activation of the p38 kinase signaling pathway and also the inhibition of NF-κB. Moreover, BB1 oral gavage treatment attenuated the DSS-induced increase in TJ permeability in the colon and promoted mucosal healing in mice (Al-Sadi et al., 2021c). A recent study also demonstrated that BB1 protected the intestinal TJ integrity from TNF-α-induced increase in TJ permeability by inhibiting the NF-κB p50/p65 pathway and MLCK gene activation through a PPAR-γ-dependent mechanism. BB1 exerts its effect *via* the TLR-2/TLR-6 receptor complex, and activates PPAR-γ, which in turn suppresses TNF-α-induced IKK-α activation and degradation of IκB-α both *in vitro* and *in vivo* models (Abdulqadir et al., 2024). Another study using a mouse model of DSS-induced intestinal damage, *B. bifidum* FL-228.1 (FL-228.1), showed the most significant prophylactic effect. Mechanistic analysis revealed that FL-228.1 enhanced the expression of mucin 2 and Claudin-4 in the colon. Transcriptomic and protein-protein interaction analyses suggested that its protective effects are mediated through inhibition of the NLRP3 inflammasome and activation of PPARγ and TLR-2 signaling pathways. Pre-administration of FL-228.1 significantly strengthened intestinal barrier integrity through immune modulation and enhancement of key barrier proteins (Wang et al., 2023). The beneficial effect of bifidobacteria in treating various GI disorders has also been reported in various mouse models. Bergmann et al. (2013) reported the protective effect of *B. infantis* in a mouse NEC model. Compared to dam-fed controls, mouse pups administered with *B. infantis* saw attenuated increase in intestinal permeability, preserved occludin and claudin-4 localization at TJs and decreased NEC incidence. Another species, *B. bifidum*, has been shown to improve intestinal integrity in a rat model of NEC (Khailova et al., 2009). On the other hand, *B. animalis* subspecies *lactis* was shown to be efficient in restoring gut barrier permeability in a DNBS (dinitrobenzene sulfonic acid) induced low-grade inflammation model in mice. *Bifidobacterium animalis* subspecies *lactis* protected intestinal barrier by normalizing the levels of several TJ proteins, in particular claudin-4 and also by restoring the helper T cells Th1/Th2 ratio balance in colonic goblet cell population (Martin et al., 2016). Srutkova et al. (2015) showed that *Bifidobacterium* can ameliorate acute DSS-induced colitis in mice. The probiotic strain *Bifidobacterium longum* ssp. *longum* CCM 7952 (Bl 7952), but not Bl 372, maintained expression of TJ proteins and reduced serum FITC-dextran levels, correlating with reduced disease activity index (DAI) (Srutkova et al., 2015). Numerous studies with animal models and human patients of alcoholic liver disease have shown that prolonged consumption of alcohol causes imbalance in gut microbiota and microbial metabolites, leading to defects in intestinal epithelial barrier (Iyer and Vaishnava, 2016). It is hypothesized that this increased gut permeability leads to higher LPS concentration in portal blood circulation where LPS binds to TLR-4 and activate NF-κB, which in turn stimulates expression of pro-inflammatory cytokines (Zhou and Zhong, 2017). *Bifidobacterium* has been reported to improve the paracellular permeability in Caco-2 monolayers treated with LPS by significantly decreasing the production of pro-inflammatory cytokines (such as IL-6 and TNF-α) and upregulating TJ protein (occludin, claudin-3 and ZO-1) expression and localization (Ling et al., 2016).

It is worth noting that there are lines of evidence that show the effect of *Bifidobacterium* is strain-specific, and hence interaction of different *Bifidobacterium* species with host cells may have distinct effects (Braga et al., 2017). For instance, supplementation of *B. longum* showed a decrease in the expression of genes encoding pro-inflammatory cytokines (Furrie, 2006), while ingestion of *B. animalis* subsp. *lactis* caused an increase in the anti-inflammatory cytokine TNF-α and in phagocytic activity (Arunachalam et al., 2000). These studies caution that the beneficial effects of one probiotic strain cannot be applied to other species or even subspecies of the same genus. Bacteria-free conditioned media of *B. infantis* was found to protect against NEC by suppressing the activation of NF-κB *via* preserved IκB expression. The conditioned media also suppressed the production of the pro-inflammatory cytokine TNF-α, a downstream target of NF-κB pathway (Shiou et al., 2013), and also prevented the IL-1β-induced increase in intestinal permeability, similar to the *L. acidophilus* effect (Guo et al., 2017). Purified galactooligosaccharide, derived by the galactosyltransferase activity of B. *bifidum* (using lactose as the substrate) was shown to reduce the adhesion and invasion of *Salmonella enterical serovar* and *S. thyphimurium* both *in vitro* and *in vivo* (Searle et al., 2010).

Although a plethora of studies have proven the health-promoting activities of bifidobacteria species, especially their role in maintaining the intestinal epithelial TJ barrier, the underlying molecular mechanisms remains unknown. Major struggles behind this include 1) the strain-specific activity of *Bifidobacterium,* which makes it difficult to define a specific pathway of action; 2) the complex interaction of bifidobacteria with human host cells and other macrofloral members of the gut; 3) the notoriously recalcitrant nature of bifidobacteria to genetic modification. The development of effective molecular tools and more focused studies is expected to unravel the molecular mechanisms that explain how bifidobacteria interact with their human host and exert their beneficial effects.

## Saccharomyces boulardii

Earlier studies showed the probiotic yeast *S. boulardii* protected the EPEC-induced decrease in TER and increase in mannitol flux in T84 monolayers; however, *S. boulardii* did not alter the TJ barrier function in healthy T84 cells. The protective effect of *S. boulardii* was mediated by the preservation of ZO-1 and by inhibiting ERK1/2 signaling pathway (Czerucka et al., 2000). Garcia Vilela *et al.* showed that *S. boulardii* decreased the intestinal permeability in CD patients by 33% at the end of third month compared to patients with no probiotic treatment (Garcia et al., 2008). Furthermore, *S. boulardii* CNCM I-745 has been shown to protect against pathogen-induced TJ barrier dysfunction (Terciolo et al., 2019). Dahan *et al.* showed that coincubation with S. *boulardii* helped to maintain the epithelial barrier integrity, while preincubation was required to significantly reduce IL-8 secretion. *Saccharomyces boulardii* preserved barrier function and inhibited EHEC-induced inflammation by blocking MLC phosphorylation and suppressing the NF-κB and MAPK signaling pathways in T84-infected cells (Dahan et al., 2003). Another study examined the effects of S. *boulardii* on *Shigella flexneri* infection using both *in vitro* and *in vivo* models of human intestinal epithelium. During infection, S. *boulardii* enhanced barrier integrity by upregulating the TJ protein ZO-2 and reducing activation of ERK, Jun N-terminal kinase (JNK), and NF-κB signaling pathways. A cell-free S. *boulardii* supernatant reproduced these anti-inflammatory effects. *Saccharomyces boulardii’s* anti-inflammatory effect was confirmed in a human fetal intestinal xenograft model, where the yeast strain alleviated damage and inflammation inflicted by *S. flexneri*, but was not able to prevent infection (Mumy et al., 2008).

## Probiotic combination

Blackwood *et al.* showed the effect of probiotics *L. plantarum* and *L. rhamnsosus* on intestinal TJ barrier function in both *in vitro* and *in vivo* models of NEC. They added *L. plantarum* and *L. rhamnsosus* to the apical surface of Caco-2 cells at a concentration of 10^7^ CFU/mL for 5 h. The pretreatment of *L. plantarum* and *L. rhamnsosus* increased TER and decreased dextran flux across monolayers compared to control groups. In addition, pretreatment with both *L. plantarum* and *L. rhamnsosus* attenuated the LPS and EGTA-induced damage to the TJ barrier by modulating the expression of ZO-1. *L. rhamnsosus* appeared to provide a greater degree of protection against EGTA- or LPS-mediated injury than did *L. plantarum*. In a rat model of NEC infected with *Cronobacter sakazakii* (CS), the combination of *Lactobacillus* spp. caused an increase in intestinal permeability, which is contradictory to the *in vitro* model; however, pretreatment with the combined probiotics prior to CS infection did not increase in intestinal permeability but protected against intestinal injury. The authors concluded that the probiotics themselves may be harmful to the intestinal epithelial cells. Thus, the clinicians should be cautious in using specific probiotics (Blackwood et al., 2017). A recent study demonstrated that the BWI mix - a multi-species probiotic mixture comprising eight live strains and one heat-treated strain [*Lactiplantibacillus plantarum* LM1001 (KCCM 42959) (47.82%), *Limosilactobacillus reuteri* LM1071 (KCCM12650P) (19.79%), *Bifidobacterium animalis* ssp. lactis HEM 20-01 (KCTC 14143BP) (6.59%), *B. animalis* ssp. lactis LM1017 (KCCM12629P) (6.60%), *Lactococcus lactis* LM1009 (KCCM 80146) (8.24%), *B. longum* LM1024 (KCCM 80145) (0.25%), *Limosilactobacillus fermentum* HEM 1036 (KCTC 13978BP) (1.65%), and *Streptococcus thermophilus* LM1012 (KFCC 11771P) (0.82%)] and heat-killed material from *Lactiplantibacillus plantarum* LM1004 (KCCM 43246) (8.24%) - effectively supported intestinal barrier function. On an *in vitro* co-culture model of differentiated Caco-2 and THP-1 cells, the BWI mix preserved epithelial barrier integrity by maintaining occludin protein levels and activating the AMPK signaling pathway, which is critical for TJ assembly. Under LPS-induced inflammatory conditions, the BWI mix also reduced proinflammatory cytokine gene expression by inhibiting the NFκB signaling pathway. TER measurements confirmed that BWI mix prevented increased epithelial permeability in a dose-dependent manner (Han et al., 2023). Another double-blind study showed a significant reduction in antibiotic associated diarrhea in infants treated with a commercial probiotic formula containing *B. bifidum* and *Streptococcus thermophiles* (Correa et al., 2005).

A comparative study of the protective effect against *Salmonella* infection between probiotic strains of Lactobacilli (*L. acidophilus* CRL 730, *L. bulgaricus* CRL 423 and *L. casei* CRL 431) suggested that only *L. casei* CRL 431 can protect against *Salmonella* by increasing intestinal barrier function and decreasing local inflammation. Although the authors did not directly measure the effect of any of the three probiotic Lactobacilli on the intestinal TJ barrier, they suggested that the protective effect of *L. casei* CRL 431 was due to the preservation of the barrier function and attenuation of inflammation. They also suggested that when a probiotic strain exhibits immunomodulatory properties, this does not guarantee a protective effect against other pathogens and strain‐specific effects might be vital for a probiotic to protect against certain entero-pathogens (Castillo et al., 2013).

Hummel *et al.* demonstrated the effect of four probiotic *Lactobacillus* species, *L. acidophilus, L. fermentum, L. gasseri,* and *L. rhamnosus*, on intestinal TJ barrier function in T84 monolayers and found that *L. acidophilus, L. fermentum* and *L. gasseri* but not *L. rhamnosus* caused an increase in TER (Hummel et al., 2012). The increase in TER was mediated by phosphorylation of adherence junction proteins E-cadherin and β-catenin and increased expression of PKC isoforms, suggesting a different mechanism of action on the intestinal TJ barrier function by different probiotic strains (Hummel et al., 2012).

Following this study, Madsen *et al.* determined the efficacy of the probiotic combination, VSL#3, (containing 9 × 10^10^ colony-forming units (cfu)/g of viable, lyophilized bifidobacteria (*B. longum, B. infantis,* and *B. breve*), 8 × 10^10^ lactobacilli (*L. acidophilus, L. casei, L. delbrueckii* subsp. *L. bulgaricus*, and *L. plantarum*), and 20 × 10^10^ of *Streptococcus salivarius* subsp. *thermophilus*) in the treatment of colitis in the interleukin (IL)-10 -10-deficient mouse and in modulating the intestinal TJ barrier function (Al-Sadi et al., 2021c). Previously, VSL#3 has shown efficacy in the maintenance treatment of pouchitis, UC, and in preventing postoperative recurrence of CD (Pronio et al., 2008; Chapman et al., 2007; Chapman et al., 2006; Karimi et al., 2005). Madsen’s study showed that VSL#3 treatment did not affect the colonic electrical resistance but significantly decreased the mannitol flux, whereas IL-10-deficient mice treated with VSL#3 exhibited a significant drop in colonic resistance compared to control mice and a marked increase in mannitol flux. After 4 weeks, mannitol flux was normalized in the IL-10-deficient mice treated with VSL#3, suggesting that the probiotics in VSL#3 can be present in the colon and alter colonic permeability. In the same report, it was shown that live bacteria, but not heat-inactivated bacteria, of VSL#3 can cause a 20% increase in TER in T-84. The beneficial effects of VSL#3 in animal models are most likely due to a combination of certain Lactobacilli spp. adhere to mucosal surfaces, inhibition of the attachment of other pathogenic bacteria, as well as secretion of soluble factors that enhance barrier integrity (Madsen et al., 2001). Another study showed that VSL#3 caused a 40% increase in TER over 12-h experimental period and prevented *Salmonella dublin*-induced decrease in TER in T-84. The protective effect of this probiotic mixture was found to be accompanied by a decrease in IL-8 production and mucin expression (Otte and Podolsky, 2004). Corridoni *et al.* showed that VSL#3 decreased epithelial paracellular permeability in a TNF-α-dependent manner in the ileum of pre-inflamed SAMP1/YitFc (SAMP) mice. SAMP mice displayed an inherent increase in small intestinal epithelial paracellular permeability that preceded the histologic onset of ileitis and was independent on commensal flora colonization. The SAMP strain represents a spontaneous model of chronic intestinal inflammation that resembles CD for disease location (i.e., terminal ileum), histologic features, and responds to standard therapies to Crohn’s patients. In addition, this study demonstrated that VSL#3 treatment resulted in an increase in occludin and a decrease in claudin-2 expression (Corridoni et al., 2012). Mennigen *et al.* showed that VSL#3 prevented the DSS-induced increase in colonic permeability to Evans blue, and that was associated with VSL#3 inhibition of DSS-induced decrease in occludin, ZO-1, claudin-1, -3, -4 and -5 expression (Mennigen et al., 2009).

Live bacteria of *L. plantarum* MF1298 and *L. salivarius* DC5 showed a dose-dependent increase in TER (∼40%) which was mediated by an increase in ZO-1 protein expression in filter-grown Caco-2. However, heat-killed MF1298 and DC5 showed no effect on TER, suggesting that heat denatures the surface proteins of *lactobacilli*, which are known to be involved in their adhesion to epithelial cells. Furthermore, the supernatant harboring secreted metabolites from MF1298 and DC5 did not increase TER. Collectively, these data suggested that the presence of live bacteria is required to modulate TER. Pretreatment of MF1298, but not DC5, temporarily attenuated the decrease in TER induced by pathogenic *Listeria monocytogenes* in Caco-2 (Klingberg et al., 2005).

Earlier studies by Resta-Lenert and Barret demonstrated that a combination treatment of two probiotics, *S. thermophilus* (ST) and LA, caused a small but significant increase in TER in Caco-2 and HT-29 cells. However, neither *S. thermophiles* or *L. acidophilus*, nor both strains in combination, altered permeability to paracellular marker dextran. The increase in TER was accompanied by modulation of phosphorylation of ZO-1 and occludin. Conditioned media, antibiotic-treated, and heat inactivated cultures from *S. thermophiles* or *L. acidophilus*, all failed to increase TER or decrease permeability to dextran. It is concluded that live *S. thermophiles* and *L. acidophilus* improve epithelial barrier properties and act as a potential mechanism contributing to their beneficial effect *in vivo* (Resta-Lenert and Barrett, 2003). In a follow-up study, *S. thermophiles* and *L. acidophilus* prevented the TNF-α and IFN-γ-induced drop in Caco-2 and HT-29 TER and increase in intestinal permeability (Resta-Lenert and Barrett, 2006). Another study showed that exopolysaccharide produced by *S. thermophilus* MN-BM-A01 prevented the LPS-induced drop in TER and increase in dextran flux in Caco-2 (Chen et al., 2019).

Previous studies by Gotteland et al. showed the combinational effect of *L. rhamnosus*, *L. acidophilus* and *L. helveticus* on gastric and intestinal permeability in healthy human volunteers. Indomethacin, a chronic nonsteroidal anti-inflammatory drug (NSAID), caused an increase in both gastric and intestinal permeability, live bacteria significantly reduced the alteration of gastric but not intestinal permeability induced by indomethacin (Gotteland et al., 2001). Another study showed that combinational treatment of *L. helveticus* R0052 and *B. longum* R0175 had a protective effect on the myocardial infarction-induced increase in intestinal TJ permeability in rats. However, the combined treatment of these two probiotic strains did not show any effect on the intestinal barrier in control rats. It was hypothesized that these 2 probiotic strains might have inhibited the NF-κB signaling pathway and activated an anti-apoptotic pathway and TLR-2 (Arseneault-Breard et al., 2012).

## Conclusion and perspectives

The intestinal epithelial TJ barrier is a critical regulator of gut homeostasis, and its disruption is a common feature across a spectrum of GI disorders, including IBD, IBS, NEC, and pathogen-induced inflammation. Accumulating evidence from *in vitro* studies, animal models, and clinical trials supports the role of probiotics in preserving or restoring TJ integrity. Specific strains of probiotics have demonstrated protective effects on the intestinal barrier through diverse mechanisms, including upregulation of TJ proteins, modulation of inflammatory signaling pathways (e.g., NF-κB, MAPKs), inhibition of MLCK activity, and interaction with host pattern recognition receptors such as TLRs. Importantly, these effects are highly strain-specific and in some cases, dependent on the viability of the organisms, their metabolites, or cell surface components. Moreover, probiotics can exert differential effects in epithelial cells *versus* immune cells, suggesting cell–type–specific mechanisms of action. While live probiotics have shown efficacy in preclinical and clinical settings, their use in immunocompromised populations requires caution.

Despite extensive research, significant gaps remain in our understanding of the precise molecular mechanisms by which probiotics enhance TJ barrier function. An important challenge in interpreting probiotic studies on intestinal TJ barrier function is the variability observed across studies, even when the same probiotic strain is tested. Several factors may account for these discrepancies. First, genetic and metabolic differences among probiotic strains within the same species can result in divergent effects on host signaling. Second, host-related factors such as species, genetic background, and disease state can influence probiotic efficacy. Third, methodological differences, including the use of immortalized cell lines, animal tissues or primary organoids, to variations in barrier assays (TER versus flux of paracellular markers such as mannitol, dextran and inulin) can yield different outcomes. Finally, dose and time effects are rarely standardized across studies. Recognizing these sources of variability may explain why probiotic effects on TJ regulation are sometimes inconsistent. Future research should focus on identifying strain-specific signaling pathways, optimizing dosage and delivery methods, and conducting well-controlled clinical trials to validate efficacy in targeted patient populations.

In conclusion, probiotics represent a valuable and biologically plausible therapeutic approach to reinforcing the intestinal TJ barrier and mitigating barrier-related GI pathologies. Continued investigation into their molecular mechanisms and clinical utility will be essential to harnessing their full potential in the prevention and treatment of intestinal barrier dysfunction.
