# Supplementary material for: Probiotics and the intestinal tight junction barrier function
Source: Front Cell Dev Biol. 2025 Dec 1;13:1671152. doi: 10.3389/fcell.2025.1671152 (PMC12702976; doi:10.3389/fcell.2025.1671152)
Supplement: Supplementary file 1 [file Table1.docx]

**Table 1.** Mechanisms of probiotic strains on intestinal tight junction barrier function.

Legend: *Live, ^heat-killed, #supernatant

Table Abbreviations (Supplementary Sheet)

| **Probiotic bacteria** | **Cell and/or animal model** | **Treatment or disease condition** | **Associated mechanisms with TJ barrier function** | **Reported targets** | **Refs.** |
| --- | --- | --- | --- | --- | --- |
| ***Lactobacillus (L.)*** | | | | | |
| **L. acidophilus 1*  (LA1) | Caco-2; mouse | TNF-ɑ;  i.p. injection TNF-ɑ | Prevent TNF-ɑ-induced ↓ TER  TLR-2-dependent inhibition of NF-ĸB pathway & MLCK activity | ↑ IKK-a, PI3K, TLR-2  ↓ NF-ĸB p50/p65, MLCK | (48) |
| *L. paracasei* | Mouse | *E. coli* O8- induced diarrhea | Prevent *E. coli* O8-induced diarrhea  ↑ relative body weight  ↓ diarrhea rate & index | ↑ SCFA-producing bacteria  ↓ serum DAO, zonulin, IL-6, IL- 1β, TNF-ɑ, NF-ĸB p65, MLCK, MLC-2 | (50) |
| *L. rhamnosus GG*  fermented milk | Mouse | DSS | Prevent intestinal inflammation via activation of EGFR  ↓ inflammation & injury | ↑ AKT, EGFR, p40,  p75 | (55) |
| **L. amylophilus* D14 | Caco-2 | *E. coli* K88, *S. typhi* | Prevent E. coli K88 or S. typhi- induced ↓ TER  ↓ dextran flux | ↑ CDH-1, CLDN-1, ZO-1  ↓ ERK, IL-8, JNK | (57) |
| *L. acidophilus*  NCFM (SLP*)* | HT-29 | E. coli, S. typhi | Mitochondria-mediated protection against E. coli or S. typhi-induced apoptosis  Inhibit adhesion & invasion of E. coli & S. typhi  ↓ intracellular Ca^2+^ flux | ↑ MMP  ↓ CASP-3, CASP-9 | (59) |
| *L. acidophilus* (SLP) | Caco-2 | TNF-ɑ | ↑ TER  Protect IEC permeability | ↑ OCLN, ZO-1  ↓ IL-8, NF-ĸB | (63) |
| **L. acidophilus 1*  (LA1) | Caco-2; mouse | DSS (mouse) | TLR-2-dependent protection of TJ barrier function & IEC permeability  ↑ TER  ↓ inulin & dextran flux | ↑ TLR-1, TLR-2, TLR-6 | (64) |

| **L. acidophilus* W37 | Caco-2 | S. typhi | Prevent S. typhi-induced barrier disruption  ↑ TER | ↑ OCLN, CLDN-4, CLDN-15, CLDN- 16  ↓ IL-8 | (65) |
| --- | --- | --- | --- | --- | --- |
| *L. acidophilus* (CM) | Caco-2 | IL-1β | ↑ TER  ↓ dextran flux | ↑ OCLN, CLDN-1  ↓ NF-ĸB | (66) |
| *^#L. acidophilus* LB | HT-29 | Aspirin | Prevent aspirin-induced alterations in TJ permeability | ↑ ZO-1 | (67) |
| **L. acidophilus* | Mouse | *S. typhi* | Protect TJ barrier function Inhibit Notch pathway | ↑ MATH-1  ↓ IL-1β, DLL-1, DLL-4, HES-1 | (68) |
| *L. acidophilus*  LAB20 | Caco-2, HT-29 | LPS | ↑ TER | ↓ IL-8 | (69) |
| **L. casei* Lcr35 | Caco- 2/PBMC  co-culture | LPS | ↑ TER | ↓ IL-8, MCP-1 | (70) |
| *L.casei* ATCC 393 | PMMC, IPEC-J2;  mouse | ETEC | Prevent ETEC-induced intestinal mast cell activation & barrier dysfunction  ↑ TER  ↓ dextran flux | ↑ ZO-1, OCLN, CLDN-1, TLR-2, TLR-4 | (71) |
| *L.casei* DN-114 001 | Mouse | DSS | Prevent DSS-induced ↑ dextran flux | ↓ TNF-ɑ, IFN-γ | (72) |
| **L. casei* | Caco-2 | TNF-ɑ, IFN-γ | Prevent TNF-ɑ, IFN-γ--induced ↓ TER | ↑ ZO-1, TLR-2, MAPK, PI3K/p- AKT | (73) |
| *L.casei* DN-114 001 | T-84 | EPEC | Prevent EPEC-induced ↓ TER | ↑ ZO-1 | (74) |
| *L. plantarum*  WCFS1 | Caco-2 BBE | TNF-ɑ, LPS, PCSK, PDBu | TLR-2-dependent ↑ TER | ↑ ZO-1, TLR-2 | (76) |
| *L. plantarum*  ZLP001 | IPEC-2;  piglet | ETEC | ↑ TER  Improve gut microbiota | ↑ ZO-1, CLDN-1, OCLN  ↓ IL-6, IL-8, TNF-ɑ | (77, 78) |
| *L. plantarum*  NCU116 (EPS116) | Caco-2; Mouse | DSS (mouse) | STAT3-dependent ↑ TER & protection of TJ barrier function | ↑ STAT3, ZO-1, OCLN | (79) |

| *L. plantarum* (LP) | NCM460 | LP-stimulated NK cells, ETEC | LP-stimulated NK cells protect against ETEC-induced barrier dysfunction  ↑ TER | ↑ ZO-1, OCLN, TLR-2, IL-22, p- STAT-3, p-TYK-2 | (80) |
| --- | --- | --- | --- | --- | --- |
| *L. plantarum* DSM 2648 | Caco-2 | EPEC | Prevent EPEC-induced ↓ TER | ↓ ETEC adherence | (81) |
| *L. plantarum*  HY7714 | Caco-2 | TNF-ɑ | Prevent TNF-ɑ-induced ↓ TER | ↑ ZO-1, OCLN, CLDN-1  ↓ ELK-1, NF-ĸB,  MLCK, IL-6, IL-8, IL-1β | (82) |
| *L. plantarum*  MB452 | Caco-2 | N/A | ↑ TER | ↑ ZO-1, ZO-2,  OCLN, cingulin | (83) |
| *L. plantarum* LR002 | Mouse | DSS | ↑ colon length  ↓ DAI, injury severity | ↑ ZO-1, CLDN-3, OCLN, PPARg  ↓ MAPK/NF-ĸB, IL- 1β, IL-6, TNF-ɑ, MPO, SOD, MDA | (84) |
| *L.rhamnosus*  CNCM I-3690 | Caco-2; mouse | TNF-ɑ; DNBS | Prevent TNF-ɑ-induced ↓ TER;  ↓ dextran flux, injury severity | ↑ MUC-2, Treg, IL- 10, GHR, PYY, GUCA-2B  ↓ NF-ĸB | (86) |
| **L. rhamnosus GG* | Caco-2 | TNF-ɑ, IFN-γ | Prevent TNF-ɑ, IFN-γ--induced ↓ TER | ↑ ZO-1  ↓ NF-ĸB, ERK1/2, CXCL-8, CCL-11 | (87) |
| **L. rhamnosus GG* | Human enteroids & colonoids | IFN-γ | ↓ dextran flux | ↑ ZO-1, OCLN | (88) |
| **L. rhamnosus GG* | Caco-2 | Gliadin-induced  ↑ permeability | ↑ TER  ↓ lactulose flux | ↑ ZO-1, CLDN-1, OCLN  ↓ zonulin | (89) |
| **L. rhamnosus GG* | MDCK-I, T-84 | EHEC | Prevent EHEC-induced ↓ TER  ↓ dextran flux | ↑ ZO-1, CLDN-1 | (90) |
| *L. rhamnosus*  MTCC-5897 | Caco-2 | *E. coli* | Prevent E. coli-induced ↓ TER | ↑ ZO-1, OCLN | (91) |
| **^L. rhamnosus*  OLL2838 | Caco-2; mouse | TNF-ɑ; DSS | Prevent TNF-ɑ-induced ↓ TER  ↓ IEC apoptosis | ↑ ZO-1, MLCK  ↓ IL-8 | (92) |
| **L. helveticus*  ASCC 511 (LH511) | IPEC-J2 | *E. coli* LPS | Prevent E. coli-induced ↓ TER  ↓ EHEC & EIEC adhesion | ↑ ZO-1, OCLN, CLDN-1, TLR-2, TLR-4  ↓ A20, IRAK-M, TNF-ɑ, IL-6, IL-8 | (93) |
| *L. reuteri* FN041 | Mouse | HFD-induced ↑ intestinal permeability | Prevent HFD-induced ↑ intestinal permeability;  Modification of gut microbiota, SCFA production | ↑ ZO-1, OCLN, CLDN-6, plgR, REG-3g  ↓ TNF-ɑ, serum LPS & LBP,  colonic IAP | (95) |

| *L. reuteri* (LR1) | IPEC-1 | ETEC | MLCK-dependent prevention of ETEC-induced ↑ in permeability;  ↓ adhesion & invasion of coliform | ↑ MLCK, ZO-1, OCLN | (51) |
| --- | --- | --- | --- | --- | --- |
| *L. reuteri* (LR1) | IPEC-1 | ETEC | ↓ adhesion of ETEC, dextran flux | ↑ ZO-1, IL-10  ↓ IL-6, TNF-ɑ | (96) |
| **#L. reuteri* 15007 | IPEC-J2;  piglets | LPS (IPEC-J2) | ↑ TER;  Promote maturation of intestinal mucosal barrier in formula-fed piglets | ↑ ZO-1, CLDN-1, OCLN  ↓ TNF-ɑ, IL-6 | (97) |
| **L. rhamnosus* GG | IPEC-J2 | ETEC | Prevent ETEC-induced ↓ TER  ↓ ETEC adhesion | ↑ ZO-1 | (98) |
| *#*L. johnsonii* P47- HY |  |  | Prevent ETEC-induced ↓ TER  ↓ ETEC adhesion | ↑ ZO-1, HSP-27 |  |
| **#L. reuteri* P43- HUV |  |  | Prevent ETEC-induced ↓ TER Cryoprotection against intestinal damage | ↑ ZO-1, HSP-27, HSP-72 |  |
| *L. salivarius*  UCC118*,*  *L. salivarius*  CCUG38008 | Caco-2; mouse | Hydrogen peroxide; DSS | Prevent H2O2-induced ↓ TER  ↓ dextran flux | ↑ ZO-1, OCLN, CLDN-1, JAM-1 | (99) |
| *L.salivarius*  SMXD51 | Caco- 2/TC7 | *P. aeruginosa*  PAO1 | ↑ TER  Protect F-actin cytoskeleton Protect IE barrier & permeability | ↑ F-actin, IL-8, HBD-2 | (100) |
| *L. plantarum,*  *L. rhamnosus* | Caco-2; Rat pup | LPS, EGTA; NEC, CS | Prevent LPS & EGTA-induced ↓ TER  ↓ FITC dextran flux  CS-dependent ↓ intestinal injury | ↑ ZO-1 | (142) |
| *L. casei* CRL 431 | Mouse | S. typhi | Protect against Salmonella infection by ↑ intestinal barrier function & ↓ local inflammatory response | ↑ S-IgA, MCP-1, IL-6  ↓ TNF-ɑ | (145) |
| *L. acidophilus,*  *L. fermentum,*  *L. gasseri,*  *L. rhamnosus* | T-84 | EPEC | Prevent EPEC-induced ↓ TER | ↑ PKCδ, CDH-1 | (146) |
| **L. plantarum*  MF1298*,*  **L. salivarius* DC5 | Caco-2 | Listeria monocytogenes | ↑ TER | ↑ ZO-1 | (155) |
| *L. helveticus*  R0052,  *Bifidobacterium longum* R0175 | Rat | MI | ↓ dextran flux | ↓ IL-1β | (160) |
| ***Escherichia coli Nissle 1917 (EcN)*** | | | | | |
| *#EcN* | HT-29/B6,  Caco-2 | N/A | TcpC-dependent ↑ TER  ↓ mannitol flux | ↑ TcpC, PKCδ, ERK1/2, CLDN-15 | (101) |
| *#EcN* | IEC-6 | 5-FU-induced IEC damage | ↑ TER, cell viability  ↓ apoptosis | N/A | (102) |
| *#EcN* | Caco-2; mouse | TNF-ɑ/IFN-γ;  CLP-induced sepsis | Prevent TNF-ɑ/IFN-γ-induced ↓ TER | ↑ ZO-1, OCLN, CLDN-1  ↓ CLDN-2, MLCK-P-MLC pathway, NF-ĸB | (103) |

| *EcN, ECOR63, OMVs* | T-84,  Caco-2 | EPEC | Prevent EPEC-induced ↓ TER | ↑ ZO-1, OCLN, CLDN-14 | (104) |
| --- | --- | --- | --- | --- | --- |
| *EcN, E. coli*  MG1655 | Mouse | DSS | Protect against DSS-induced ↑ mucosal permeability to luminal substances  ↑ net Na^+^ absorption  ↓ permeability to Evans Blue | ↑ ZO-1 | (105) |
| ***Bacillus (B.)*** | | | | | |
| **#Bacillus subtilis*  29784 | Caco-2 | IL-1β, DON, Fla | Prevent IkB degradation & nuclear translocation of NF-ĸB  ↑ TER | ↑ ZO-1, OCLN, CLDN-1, IkB  ↓ iNOS, IL-8, IL- 1β, NF-ĸB | (106) |
| ***Bifidobacterium (B.)*** | | | | | |
| **B. bifidum* (BB1) | Caco-2; mouse | TNF-ɑ; DSS | TLR-2 & p38 kinase-dependent protection against DSS-induced colitis  ↑ TER  ↓ dextran flux | ↑ TLR-2/TLR-6, PPAR-y, p38  kinase  ↓ IKK-a, NF-ĸB p50/p65, MLCK | (47, 127) |
| *B. infantis* (CM) | Caco-2 | IL-1β | ↑ TER  ↓ dextran flux | ↑ OCLN, CLDN-1  ↓ NF-ĸB | (66) |
| *HMO-grown B. longum infantis, B. bifidum* | Caco-2, HT-29 | N/A | Prophylactic enhancement of intestinal barrier function | ↑ OCLN, JAM-A, IL-10 | (119) |
| *B.*  *bifidum* LMG13195 | PBMC,  monocyte- derived DC | LPS | Improve immune response to mucosal inflammation  Promote generation of functional Treg cells, Th17 differentiation | ↑ IL-17, IL-1β, CD80, CD86, CD25  ↓ TNF-ɑ, IFN-γ, CD1a, CD127, IL- 10 | (121) |
| *B. infantis* | Neonatal mouse | NEC | ↓ NEC severity & incidence, dextran flux | ↑ CLDN-4, OCLN | (123) |
| *B. bifidum* PRL2010 | Mouse | TNBS | Prevent TNBS-induced weight loss, improve stool consistency  ↓ DAI, colonic edema, histological colitis scores | ↑ TNF-ɑ, IL-12, CLDN-3, CLDN-5, JAM-A, ZO-1,  ↓ IL-10, CLDN-2 | (124) |
| *B. longum WU16,*  *B. bifidum WU12, WU20, WU57* | Caco-2 | TNF-ɑ | ↑ TER, metabolite production | ↑ OCLN, CLDN-1 | (125) |
| *Bifidobacterium (B.)* | Caco-2; rat | LPS; NEC | ↑ TER, ↓ dextran flux  ↓ NEC severity & incidence | ↑ ZO-1, OCLN, CLDN-3  ↓ IL-6, TNF-ɑ,  zonulin | (126) |
| *B. bifidum* FL-228.1 | Caco-2; Mouse | DSS (mouse) | ↑ TER | ↑ MUC-2, CLDN-4, PPAR-y, TLR-2  ↓ NLRP3, IL-18 | (128) |
| *B. bifidum* | Premature rat | NEC | ↓ NEC severity & incidence | ↓ IL-6, MUC-3, CLDN-3 | (129) |

| *B. animalis* ssp. lactis CNCM-I2494 | Mouse | DNBS | Protect against DNBS-induced disruption in intestinal barrier permeability via modulation of host T- cell composition | ↑ CLDN-4, OCLN, ZO-1, Th1/Th2, IL- 4, IL-5, IL-10  ↓ IL-12, IL-2, IL-4, IFN-γ | (130) |
| --- | --- | --- | --- | --- | --- |
| *B. longum spp. longum* CCM 7952  (BI 7952) | HEK293;  mouse | LPS; DSS | ↓ DSS-induced colitis severity | ↑ ZO-1, OCLN, TLR-2, NOD-2  ↓ TNF-ɑ, IL-10, IFN-γ | (131) |
| *B. bifidum NCIMB 41171* GOSs  (Bimuno(®)) | HT-29- 16E;  murine ligated ileal gut loop | S. typhi | Prevent adherence or invasion of S. typhi  ↓ S. typhi associated pathology  ↑ mucin production into lumen Protect TJ barrier function & permeability | N/A | (136) |
| ***Saccharomyces (S.)*** | | | | | |
| *S. boulardii* | T-84 | EPEC | Prevent EPEC-induced ↓ TER  ↓ EPEC-mediated apoptosis  ↓ inulin flux | ↑ ZO-1  ↓ ERK1/2 MAP,  CASP-3, tyrosine phosphorylation of p46 & p52 | (137) |
| *S. boulardii* | T-84 | EPEC | Prevent EHEC-induced ↓ TER, alteration of TJ structure & intestinal permeability | ↓ IL-8, MLC  phosphorylation, NF-ĸB, MAPK | (140) |
| **#S. boulardii* | T-84;  colonic xenograft | *Shigella flexneri* | Alleviate symptoms associated with host inflammatory response  ↓ HRP flux | ↑ ZO-2, CLDN-1  ↓ IL-8, ERK, JNK, NF-ĸB, PMN  transmigration | (141) |
| **Combination** | | | | | |
| *L. acidophilus, B. infantis, L. plantarum (CM)* | Rat pup | NEC-like injury | ↓ intestinal injury incidence, dextran flux | ↑ IkBa, ZO-1  ↓ NF-ĸB, TNF-ɑ | (135) |
| BWI formula  **Lactiplantibacillus plantarum* LM1001 (KCCM 42959)*,*  **Limosilactobacillus reuteri* LM1071 (KCCM12650P),  **B. animalis ssp. lactis* HEM 20-01 (KCTC 14143BP),  **B. animalis ssp. lactis* LM1017 (KCCM12629P),  **Lactococcus lactis* LM1009 (KCCM 80146),  **B. longum* LM1024 (KCCM 80145)*,*  **Limosilactobacillus fermentum* HEM | Caco-2, THP-1,  Caco-2- THP-1 co-  culture | LPS | ↑ TER  ↓ inflammation | ↑ OCLN, AMPK  ↓ NF-ĸB, COX-2,  TNF-ɑ, IL-1β, IL-8 | (143) |

| 1036 (KCTC  13978BP),  **Streptococcus thermophilus* LM1012 (KFCC 11771P)*,*  *^Lactiplantibacillus plantarum* LM1004 (KCCM 43246) |  |  |  |  |  |
| --- | --- | --- | --- | --- | --- |
| *VSL#3* | T-84;  mouse | IL-10 gene- deficient colitis | ↓ mannitol flux;  ↓ inflammation | ↓ TNF-ɑ, IFN-γ | (151) |
| ***(CM)*VSL#3* (PM) | T-84, HT-29 | *S. dublin* | Prevent *S. dublin*-induced ↓ TER & ↑ IL-8  ↑ cell survival | ↑ ZO-1, MUC-2, MUC-3, MUC-5AC, p42/44 MAPK, p38  ↓ IL-8 | (152) |
| (CM)*VSL#3* | SAMP  mouse | Ileitis | Stimulate TNF production & permeability changes in SAMP mice  ↑ TER | ↑ TNF, OCLN  ↓ TNF-ɑ, CLDN-2 | (153) |
| *VSL#3* | Mouse | DSS | Prevent DSS-induced ↑ apoptosis  ↓ inflammation | ↑ ZO-1, OCLN, CLDN-1, CLDN-3, CLDN-4, CLDN-5 | (154) |
| **S. thermophilus,*  **L. acidophilus* | Caco-2, HT-29 | EIEC | Prevent EIEC-induced ↓ TER | ↑ ZO-1, OCLN, EGFR | (156) |
| **S. thermophilus,*  **L. acidophilus* | Caco-2, HT-29 | TNF-ɑ, IFN-γ | ↑ TER  ↓ inflammation | ↑ CFTR, NKCC1, p38, ERK1/2. PI3K  ↓ NF-ĸB | (157) |
| *Streptococcus thermophilus* MN- BM-A01 (EPS) | Caco-2; mouse | LPS; DSS | ↑ TER, colon length, body weight  ↓ dextran flux, DAI | ↑ OCLN, CLDN-1, CDH-1  ↓ TNF-ɑ, IL-6, IFN-  γ | (158) |
