# Supplementary material for: Probiotics and the intestinal tight junction barrier function
Source: Front Cell Dev Biol. 2025 Dec 1;13:1671152. doi: 10.3389/fcell.2025.1671152 (PMC12702976; doi:10.3389/fcell.2025.1671152)
Supplement: Supplementary file 2 [file DataSheet1.docx]

Table 1 abbreviations:

AKT: protein kinase B, AMPK: protein kinase AMP-activated catalytic subunit alpha-1, BM-DC: Bone marrow-derived dendritic cells, CASP: caspase, CCL: C-C motif chemokine ligand, CDH- 1: epithelial cadherin, CFTR: cystic fibrosis transmembrane conductance regulator, CLDN: claudin, CLP: cecal ligation puncture, CM: bacteria-free conditioned media, COX-2: cytochrome c oxidase subunit II, CS: Cronobacter sakazakii, CXCL: chemokine (C-X-C motif) ligand, DAI: disease activity index, DLL: delta-like canonical notch ligand, DON: deoxynivalenol, DNBS: dinitrobenzene sulfonic acid, DSS: dextran sulfate sodium, E. coli: Escherichia coli, EGFR: epidermal growth factor receptor, EGTA: ethylene glycol-bis (β-aminoethyl ether)-N,N,N′,N′- tetraacetic acid, EHEC: enterohemorrhagic Escherichia coli, EIEC: enteroinvasive Escherichia coli, ELK-1: ETS transcription factor ELK-1, ERK: extracellular-regulated protein kinase, EPS: exopolysaccharide, EPEC: enteropathogenic Escherichia coli, ETEC: enterotoxigenic Escherichia coli, F-actin: filamentous actin, FITC: fluorescein isothiocyanate, Fla: flagellin, GOS: galactooligosaccharide, GHR: ghrelin, GUCA-2B: guanylate cyclase activator 2B, HBD-2: B- defensin 2, HES-1: hes family bHLH transcription factor 1, HFD: high fat diet, HMO: human milk oligosaccharides, HRP: horseradish protein, HSP: heat shock protein, IAP: intestinal alkaline phosphatase, IEC: intestinal epithelial cell, IFN-γ: interferon gamma, IκB: inhibitor of κB, IKK-a: inhibitor KB kinase a, IL: interlukin, iNOS: inducible nitric oxide synthase, IRAK-M: interleukin 1 receptor associated kinase 3, JAM-A: junction adhesion molecule-A, JAM-1: junction adhesion molecule-1, JNK: c-jun N-terminal kinase, LBP: lipopolysaccharide-binding protein, LPS: lipopolysaccharide, MAP: mitogen-activated protein, MAPK: mitogen-activated kinase-like protein, MATH-1: atonal BHLH transcription factor 1, MCP-1: monocyte chemotactic / chemoattractant protein 1, MDA: malondialdehyde, MDCK-I: Madin-Darby canine kidney, MLC: myosin light chain, MLCK: myosin light chain kinase, MMP: mitochondrial membrane potential, MPO: myeloperoxidase, MUC: mucin, NEC: necrotizing enterocolitis, NF-κB: nuclear factor kappa B, NK: natural killer, NKCC1: Na-K-Cl cotransporter, NLRP-3: NLR family pyrin domain containing 3, NOD-2: nucleotide binding oligomerization domain containing 2, OCLN: occludin, OMVs: outer membrane vesicles, PBMC: peripheral blood mononuclear cells, PCSK: Pam3- Cys-SK4, PI3K: phosphatidylinositol 3-kinase, PKCδ: protein kinase C delta, plgR: polymeric immunoglobulin receptor, PMN: polymorphonuclear leukocyte, PMMC: porcine mucosal mast cells, PDBu: phorbol 12,13-dibutyrate, PPAR γ: Peroxisome Proliferator-Activated Receptor gamma, PYY: peptide YY, REG-3γ: regenerating family member 3 gamma, SAMP: SAMP/YitFc, SCFA: short chain fatty acid, S-IgA: secretory immunoglobulin A, SLP: surface layer protein, SOD: superoxide dismutase, STAT-3: signal transducer and activator of transcription 3, S. typhi: salmonella typhimurium, TcpC: toxin-coregulated pilus secretin TcpC, TER: trans-epithelial electric resistance, Th1: type 1 T helper cells, Th2: type 2 T helper cells, TJ: tight junction, TLR: toll-like receptor, TNF-a: tumor necrosis factor-a, Treg: T-regulatory cells, TYK-2: tyrosine kinase-2, ZO: zonula occludens, 5-FU: 5-fluorouracil.
